# Kinetic modelling for concentration and toxicity changes during the oxidation of 4-chlorophenol by UV/H_2_O_2_

**DOI:** 10.1038/s41598-021-95083-7

**Published:** 2021-08-03

**Authors:** Cristian Ferreiro, Josu Sanz, Natalia Villota, Ana de Luis, José Ignacio Lombraña

**Affiliations:** 1 Department of Chemical Engineering, Faculty of Science and Technology, University of the Basque Country UPV/EHU, Barrio Sarriena S/N, 48940 Leioa, Spain; 2 Department of Mathematics and Science Didactics, Faculty of Education, Philosophy and Anthropology of Donostia-San Sebastián, University of the Basque Country UPV/EHU, Barrio Sarriena S/N, 48940 Leioa, Spain; 3 Department of Chemical and Environmental Engineering, Faculty of Engineering of Vitoria-Gasteiz, University of the Basque Country UPV/EHU, Nieves Cano, 12, 01006 Vitoria-Gasteiz, Spain; 4 Department of Chemical and Environmental Engineering, Faculty of Engineering in Bilbao, University of the Basque Country UPV/EHU, Plaza Ingeniero Torres Quevedo, 1, 48013 Bilbao, Spain

**Keywords:** Chemical engineering, Pollution remediation

## Abstract

This work develops a kinetic model that allow to predict the water toxicity and the main degradation products concentration of aqueous solutions containing 4-chlorophenol oxidised by UV/H_2_O_2_. The kinetic model was developed grouping degradation products of similar toxicological nature: aromatics (hydroquinone, benzoquinone, 4-chlorocatechol and catechol), aliphatics (succinic, fumaric, maleic and malonic acids) and mineralised compounds (oxalic, acetic and formic acids). The degradation of each group versus time was described as a mathematical function of the rate constant of a second-order reaction involving the hydroxyl radical, the quantum yield of lump, the concentration of the hydroxyl radicals and the intensity of the emitted UV radiation. The photolytic and kinetic parameters characterising each lump were adjusted by experimental assays. The kinetic, mass balance and toxicity equations were solved using the Berkeley Madonna numerical calculation tool. Results showed that 4-chlorophenol would be completely removed during the first hour of the reaction, operating with oxidant molar ratios higher than *R* = 200 at pH  6.0 and UV = 24 W. Under these conditions, a decrease in the rate of total organic carbon (TOC) removal close to 50% from the initial value was observed. The solution colour, attributed to the presence of oxidation products as *p*-benzoquinone and hydroquinone, were oxidised to colourless species, that resulted in a decrease in the toxicity of the solutions (9.95 TU) and the aromaticity lost.

## Introduction

Chlorophenols are catalogued as one of the priority pollutants (Clean Water Act and by European Directive 2013/39/EU) according to the United States Environmental Protection Agency (US EPA)^1–3^ because they are toxic and potentially carcinogenic compounds^2^. They are considerate one of the most important classes of water contaminants due to they are difficult to remove from the environment^2–5^ and they can appear in drinking water when hypochlorite reacts during water disinfection^4^. Thus, they are of great concern.

It have been selected 4-chlorophenol as the representative member of this family of harmful compounds. Levels of 4-chlorophenol have been reported as ranging from 150 µg L^−1^^6^ to 100–200 mg L^−1^^7,8^ in contaminated environments. ^10,11^.

Advanced oxidation processes (AOPs) could help in the effective removal of these recalcitrant compounds from wastewater^9–11^. However, the cost-inefficiency of these methods limits the practical application of these technologies^12,13^. Moreover, persistent byproducts produced during the oxidation process can be released into the environment^14,15^. Thus, AOPs may be made suitable for industrial applications, if they are adequately integrated with the biological processes by combined treatments. Thus, the complete removal of water pollutants by AOPs could be combined by more environment friendly treatments leading easily biodegradable species. This propose considers that the biological oxidation process could be the last step into a combined oxidation process that results in the complete oxidation of organic load^16,17^.

Thus, it is important to develop models that can predict the toxicities of treated solutions for developing integration strategies involving AOPs and biological processes. This tool can be used for the determination of the operating conditions necessary to reduce the toxicity of the system. In this way, it is possible to achieved a water toxicity level that allow the implantation of biological processes where occur the complete degradation of the remaining pollutant load, developing in this way, a cost-effective, integrated wastewater treatment method.

The photochemical degradation of chlorophenols is a well documented process (see Supplementary Table S1). Çatalkaya et al.^18^ reported that the UV/H_2_O_2_ process could effectively reduce the toxicity of the system. This process can also improve biodegradability, decrease the intensity of the colour, and assist in the removal of micropollutants. The use of UV/H_2_O_2_ is more advantageous than semiconductor photocatalysis due to economic and feasible to implement in an industrial installation. Moreover, the use of a catalyst can lead to some operation problems such as solids management, separation and contamination in the treated water. Other effects such as screening, caused by the presence of suspended solids in industrial wastewaters would lead to a reduction of the efficiency of UV light.

The photolytic decomposition via bond cleavage occurs via a radical mechanism. The hydroxyl radicals (in the presence of oxidants such as H_2_O_2_) take part in the process as follows^19,20^:

1 $${\text{H}}_{{2}} {\text{O}}_{{2}} \mathop{\longrightarrow}\limits^{hv}{2}\,{\text{HO}}^{ \cdot } .$$

The decomposition of H_2_O_2_ can be photoinduced by the Haber–Weiss radical mechanism. The propagation steps are as follows:

2 $${\text{H}}_{{2}} {\text{O}}_{{2}} { + }\;{\text{HO}}^{ \cdot } \to {\text{H}}_{{2}} {\text{O}}\;{ + }\;{\text{HO}}_{{2}}^{ \cdot } ,$$

3 $${\text{HO}}_{{2}}^{ \cdot } { + }\;{\text{H}}_{{2}} {\text{O}}_{{2}} \to {\text{H}}_{{2}} {\text{O}}\;{ + }\;{\text{O}}_{{2}} { + }\;{\text{HO}}^{ \cdot } .$$

Finally, different termination reactions take place through radical recombination reactions:

4 $${\text{HO}}^{ \cdot } + {\text{HO}}^{ \cdot } \to {\text{H}}_{{2}} {\text{O}}_{{2}},$$

5 $${\text{HO}}^{ \cdot } + {\text{HO}}_{{2}}^{ \cdot } \to {\text{H}}_{{2}} {\text{O}}\;{ + }\;{\text{O}}_{{2}} ,$$

6 $${\text{HO}}_{{2}}^{ \cdot } { + }\;{\text{HO}}_{{2}}^{ \cdot } \to {\text{H}}_{{2}} {\text{O}}_{{2}} { + }\;{\text{O}}_{{2}} .$$

At the same time, dissociation equilibria of the organic compound itself and of the various intermediates formed, such as hydroxyl, hydroperoxide radicals, etc., according to the following:

7 $${\text{H}}_{{2}} {\text{O}}_{{2}} \to {\text{ HO}}_{{2}}^{ - } + {\text{ H}}^{ + } ,$$

8 $${\text{HO}}_{{2}}^{ \cdot } \to {\text{ H}}^{ + } {\text{ + O}}_{{2}}^{ - } .$$

Like other phenolic compounds, chlorophenols could be degraded to toxic byproducts during the initial stages of the oxidation pathway^3,21^, checking that the oxidation intermediates generated could be more toxic than the own parent pollutant (for example quinone like compounds). The formation of these byproducts could be attributed to direct photolysis or the use of low oxidant dosages^22,23^. Then, the efficiency of the oxidant process is generally determined by recording the oxidant dosage per pollutant load containing water. Thus, special attention should be paid to remove completely these toxic intermediates achieving acceptable water quality levels^24^. Consequently, it is necessary operating with excess of oxidant ratios (or an over stoichiometric addition of the oxidant) to avoid the formation of these refractory intermediates, considering that high amounts of oxidant can produce counter-effects leading to increased treatment costs. In this way, the cost-effectiveness of degradation process could be realised by optimising the amount of oxidant to reduce the wastewater toxicity.

Thus, the aim of this work consist of developing a kinetic model based on the oxidant dosage effect allow to predict the toxicity of 4-chlorophenol aqueous solutions oxidised by UV/H_2_O_2_. Consequently, the water toxicity and the formation of the different reaction intermediates were analysed during the oxidation of 4-chlorophenol. The kinetic model developed grouping three lumps of degradation byproducts as a function of their toxicity degree. The photolytic and kinetic parameters characterising each lump were adjusted in the proposed oxidation assays, obtaining a kinetic model that predict the water toxicity as a function of oxidant dose and the UV exposure time.

## Results and discussion

### Effect of oxidant dosage on water-quality parameters

Several experiments of 4-chlorophenol oxidation by H_2_O_2_/UV were carried out at pH 6.0, varying the molar ratios of hydrogen peroxide to 4-chlorophenol between R = 0–400 mol H_2_O_2_ /mol 4-chlorophenol. The objective was to analyse the effect of the oxidant in the oxidation process, where the parameters considered as water quality indicators were the pollutant concentration (C, mg L^−1^), total organic carbon (TOC, mg L^−1^), colour (AU) and loss of aromaticity (AU).

**Figure 1:**
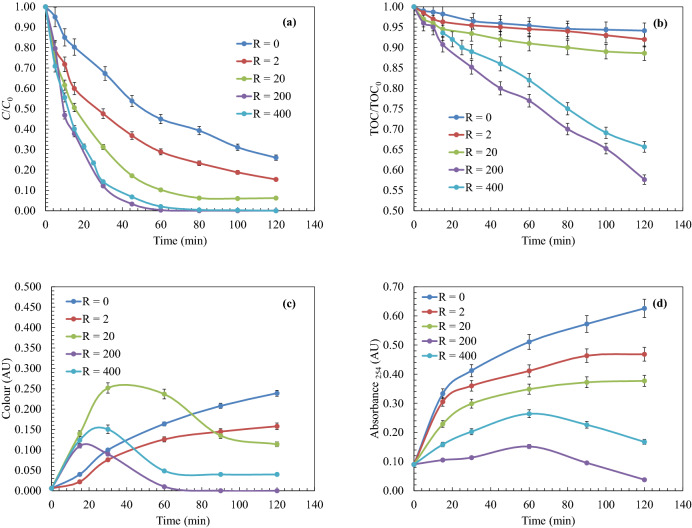
Study of the effect of peroxide dose during the UV/H_2_O_2_ oxidation of 4-chlorophenol. (**a**) Primary oxidation of 4-chlorophenol; (**b**) Mineralisation; (**c**) Colour induction; (**d**) Aromatic ring rupture. Experimental conditions: *T* = 25 ºC; *C*_0_ = 200 mg L^−1^; pH = 6.0; Agitation = 700 rpm; *V*_reac_ = 1.7 L.

Figure 1a shows that 4-chlorophenol would be completely removed from the system, during the first hour from the start of the reaction, operating with oxidant molar ratios higher than *R* = 200. Under these conditions, a decrease in the rate of TOC removal close to 50% from the initial value was observed (Fig. 1b). These results agrees well with the results (*R* values in the range of 150–375) reported by Ec et al.^25^. Operating under conditions of lower hydrogen peroxide concentration (*R* < 200), an insufficient amount of hydroxyl radicals is generated, being the photolytic pathway that dominates the radical pathway^26^. As the molar ratio of H_2_O_2_ to 4-chlorophenol increases, large amounts of the oxidant radicals are generated that assists the effective degradation of 4-chlorophenol. With the dose ratio of H_2_O_2_ used in this work, as shown in Fig. 1, primary degradation (100%) and mineralisation rate (45% of TOC removal) are achieved, in the same order of variety advanced oxidation techniques, including heterogeneous photocatalytic variants; i.e. with heterojunction^27,28^ and hybrid biomaterials^29^.

Under conditions of high reactant doses (*R* > 200), hydroxyl radicals were produced in large amounts. The excess H_2_O_2_ was converted into the hydroperoxyl radical, which exhibits less oxidative power than the hydroxyl radical (Eqs. 2, 7, 8). Additionally, the excess of peroxide was consumed by water and recombination reactions^30^. The solution colour changes during the degradation of 4-chlorophenol (Fig. 1c). Different water colours (purplish-red, reddish-orange, orange, yellow, red-brown, and light yellow) of 4-chlorophenol aqueous solutions oxidised by AOPs have been previously reported^23,31^, checking that water colour could be attributed to the presence of degradation by products generated during the oxidation process. The analyses carried out showed that the main by products formed during the oxidation pathways would be *p*-benzoquinone and hydroquinone^32^. The presence of these species would demonstrate that the colour formation is due to the formation of new chromophore groups in benzene rings, causing the generation of quinones and their derivatives^33^. Operating at *R* = 200, colour compounds were oxidized to colourless species, remaining in minor amounts, that resulted in a decrease in the toxicity of the solutions.

The decreased in the loss of aromaticity, analysed at 254 nm, described in Fig. 1d could be attributed to the rupture of the aromatic rings and could indicate the degradation of the aromatic compounds. The loss of aromaticity decreases when *R* = 200, checking that the aromatic rings ruptured and chromophore groups degraded resulting in more favourable oxidation conditions.

### Degradation pathway of 4-chlorophenol

**Figure 2:**
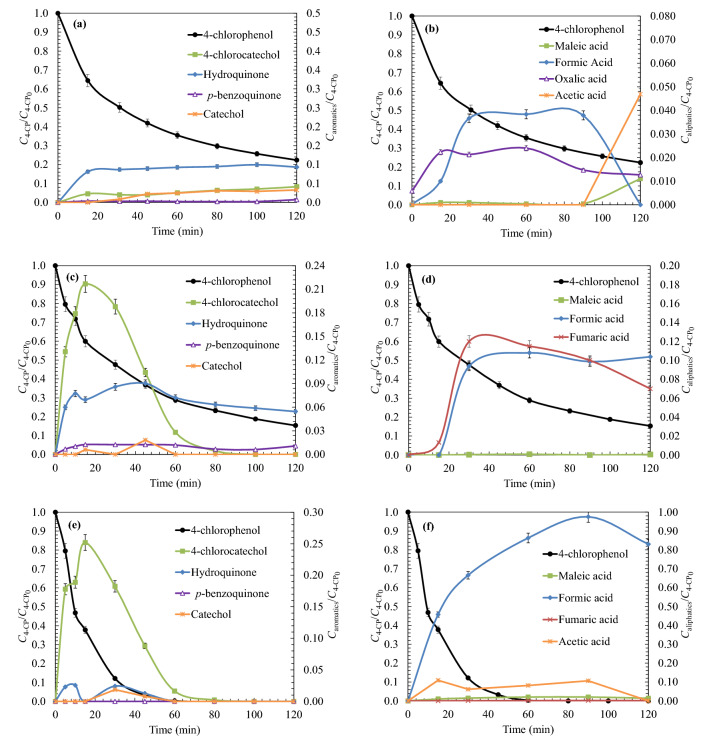
Analysis of the main degradation intermediates (left: aromatic compounds; right: aliphatic oxidation products) produced during the oxidation of 4-chlorophenol (UV/H_2_O_2_ process) under different peroxide doses: (**a**)/(**b**) *R* = 0 ; (**c**)/(**d**) *R* = 2; (**e**)/(**f**) *R* = 200. Experimental conditions: *T* = 25 ℃; *C*_0_ = 200 mg L^−1^; pH = 6.0; Agitation = 700 rpm; *V*_reac_ = 1.7 L.

Prior to the development of the kinetic model, the pollutant degradation compounds were analysed. Figure 2 shows the degradation of 4-chlorophenol, as well as the formation of their main oxidation intermediates. Results show that the oxidation of 4-chlorophenol leads to the formation of intermediate species of aromatic nature such as 4-chlorocatechol, catechol, hydroquinone and *p*-benzoquinone, as well as the formation of aliphatic species, such as maleic, fumaric, formic and acetic acids. The results obtained allow to verify that the action of UV light is capable of degrading the 4-chlorophenol rings to dihydroxylated rings (Fig. 2a). However, UV light did not prove effectiveness during the degradation of dihydroxylated intermediates to quinone-like compounds and carboxylic acids (Fig. 2b).

Thus, with the aim of degrade 4-chlorophenol until achieve higher oxidation levels, it is necessary to combine UV light with the action of an oxidant (hydrogen peroxide). Figure 1c,e show that the main degradation pathway of 4-chlorophenol evolves towards the formation of chlorosubstituted intermediates (chlorocatechol). On the other hand, once the aromatic intermediates have been degraded until carboxylic acids, it can be checked that using oxidant ratios of *R* = 200, 4-chlorophenol was completely degraded until formic acid.

**Figure 3:**
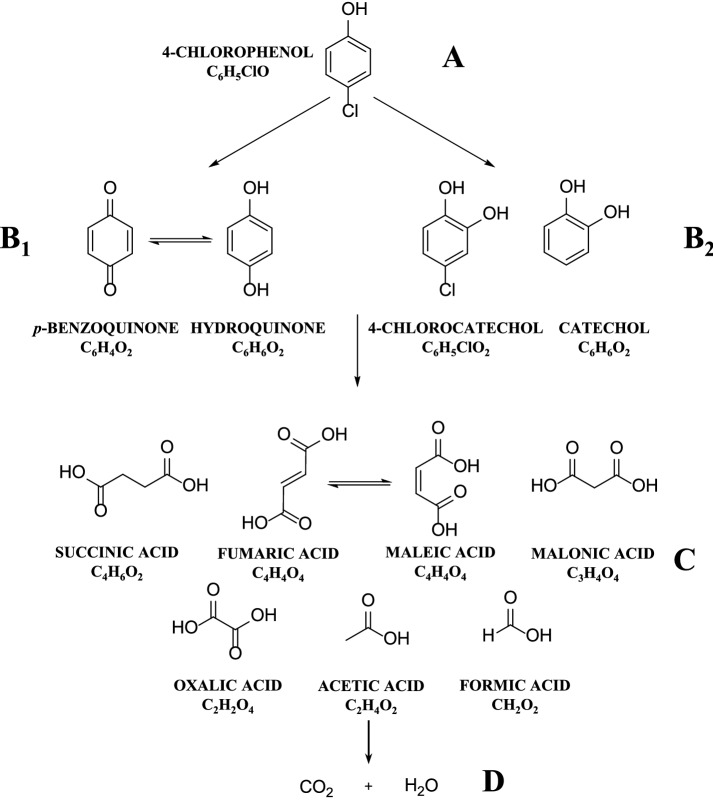
Proposed oxidation pathway for the oxidation of 4-chlorophenol via UV/H_2_O_2_ process.

The analysis performed suggests two possible reaction pathways^34^. In the first, the hydroxyl radicals attacks the aromatic rings in the *ortho*-substituted position, and displaces hydrogen, which promotes the formation of 4-chlorocatechol. In the second, the attack of hydroxyl radicals can occur in the *para*-substituted position, which cause the displacement and substitution of the chloride ion, causing the formation of hydroquinone, which is oxidised to benzoquinone (see Fig. 3).

### Kinetic modelling of the reaction intermediates pathway

**Figure 4:**
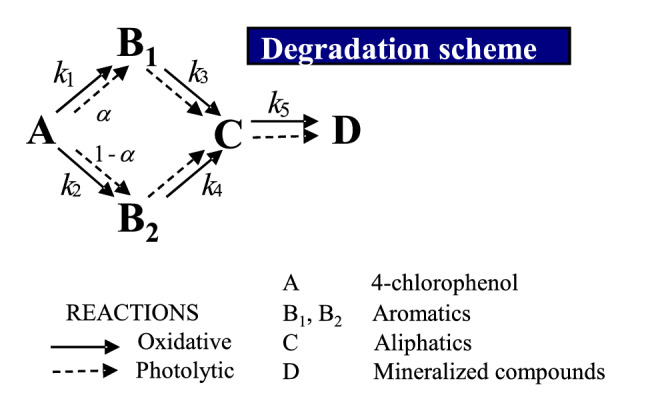
Proposed parallel reaction model for the oxidation of 4-chlorophenol using the UV/H_2_O_2_ technology.

Determination of the reaction kinetics of such complex oxidation processes can be simplified using a lumped model where all the species exhibiting similar characteristics are grouped. Thus, each group was regarded as a unique compound^35^. The contributions of different oxidation intermediates towards the total toxicity of the system depend on their individual toxicities. Then, all the oxidation intermediates do not contribute equally to the total toxicity of the system. The oxidation by products are grouped into five main groups based on their toxicity. The lumped model was constructed following previously reported protocols^24^. The mechanisms proposed by Kusic et al.^36^ and Gomez et al.^3^ have been presented in Fig. 4.

A common photolytic process forms the basis of the first stage of the oxidation process (4-chlorophenol oxidation; assigned as A). Direct photolysis promotes hydroxylation and dechlorination reactions. The highly toxic *p*-benzoquinone (*EC*_50_ = 0.035 mg L^−1^) and/or hydroquinone (*EC*_50_ = 0.088 mg L^−1^) intermediates^37^, and comparatively less toxic 1,2,4-trihydroxybenzene (*EC*_50_ = 1.03 mg L^−1^) and/or resorcinol (*EC*_50_ = 9.02 mg L^−1^) were produced in small amounts^35^.

The hydroxyl radicals turned the oxidation process via another oxidation pathway that results in the formation of other substances like hydroquinone. Chlorinated compounds and *p*-benzoquinone were also formed during the process. Aromatic compounds of varying toxicities were formed in the first step of the oxidation process. Group B_1_ consists of *p*-benzoquinone and hydroquinone. These compounds are believed to contribute the most maximum towards the global toxicity of treated wastewater as these compounds are abundant^24^. Several researchers have reported that condensation products were formed within minutes (from the start of the reaction) in the absence of oxidants (or under conditions of oxidant deficiency). The majority of the by products formed, were two-ring aromatic chlorinated compounds such as chlorinated hydroxylated biphenyls, dichlorodiphenyl ethers, and dichlorodibenzodioxins. These chlorinated compounds significantly raise the toxicity of the system^22^. In this case, water appeared yellow–brown, indicating the presence of these chromophoric toxic compounds with high molecular weight^32^.

The aromatic 4-chlorocatechol and catechol compounds formed as by products via the radical pathway are members of the B_2_ group. These compounds are less toxic than the parent compounds. In the next step of oxidation, aromatic compounds were converted to unsaturated acids (fumaric acid and maleic acid) that are subsequently converted to saturated carboxylic acids (formic acid and acetic acid). Among these, formic acid, acetic acid, fumaric acid, and maleic acid are members of group C. The constituents of this group were less toxic than the constituents of the previously described groups. Further oxidation results in CO_2_ mineralisation and formation of water (group D)^36,38^.

The change in the concentrations of the species of each group over time and the variation in toxicity over time can be determined using a kinetic model. The model was proposed by studying the reaction mechanism presented in Fig. 4. Various factors were considered for developing the kinetic model:

- Three groups (lumps) of oxidation intermediates (B_1_, B_2_, and C) were defined. Group B_1_ primarily consisted of hydroquinone, *p*-benzoquinone, and some unknown compounds (of similar structure) that exhibited high toxicity^24^. Groups B_2_ and C consisted of compounds that exhibited lower toxicity levels. The toxicity levels and the constituents were experimentally determined. Group B_2_ consisted of 4-chlorocatechol and catechol and group C consisted of various carboxylic acids (acetic acid, oxalic acid, formic acid, succinic acid, malonic acid, maleic acid, and fumaric acid).
- The degradation of 4-chlorophenol proceeds via a two ways parallel reaction mechanism. The mechanism has been previously reported by Azevedo et al.^39^ (Fig. 4). B_1_ and B_2_ are the reference lumps of each route.
- The degradation of each group proceeds via the photolytic (zero-order kinetic (*k*_i_)) and radical (pseudo-first-order kinetic (*k*’_i_)) pathways.
- The average toxicity (*EC*_50_), photolytic parameters, and kinetics constants have been determined for each lump. The experimentally obtained values of toxicity and concentration were fitted using the appropriate analysis model.

The degradation of each group versus time is described by Eq. (9), which considers the contributions of the direct photolysis and hydroxyl-radical attack processes^3,35,40^. The relation can be expressed as follows:

9 $$- \frac{{{\text{d}}C_{{\text{i}}} }}{{{\text{d}}t}} = I_{{0}} \times \Phi_{{\text{i}}} \times F_{{\text{i}}} \times \left( {{1} - {\text{exp}}\left( { - {2}{\text{.303}} \times L \times \sum {\left( {\varepsilon_{{\text{j}}} \times C_{{\text{j}}} } \right)} } \right)} \right) + \overline{k}_{{\text{i}}} \times C_{{\text{i}}} \times C_{{{\text{HO}}^{ \cdot } }},$$

where $\overline{k}_{{\text{i}}}$ is the rate constant of a second-order reaction involving the hydroxyl radical in group i, *Φ*_i_ denotes the quantum yield of lump i, *C*_HO_· denotes the concentration of the hydroxyl radicals, and *I*_0_ represents the intensity of the emitted UV radiation. *F*_i_ (incident radiation) denotes the radiation absorbed partially by each group. This can be expressed by Eq. (10) as follows:

10 $$F_{{\text{i}}} = \frac{{\sum {\left( {\varepsilon_{{\text{j}}} \times C_{{\text{j}}} } \right)_{{\text{i}}} } }}{{\sum {\varepsilon_{{\text{j}}} \times C_{{\text{j}}} } }} .$$

Equation (9) could be simplified if it was assumed that the reaction between hydroxyl radicals and 4-chlorophenol is a pseudo-first-order reaction (*k*’_i_)^19,41^. Under this condition, mass balance equations can be formulated for 4-chlorophenol (group A). The other four degradation products are grouped (groups B_1_, B_2_, C, and D) based on the total organic carbon (TOC) values. The simplified equations can be expressed as follows:

11 $$\frac{{{\text{d}}C_{{\text{A}}} }}{{{\text{d}}t}} = - I_{{\text{A}}} \times \Phi_{{\text{A}}} \times F_{{\text{A}}} - \;k^{\prime}_{{1}} \times C_{{\text{A}}} - k^{\prime}_{{2}} \times C_{{\text{A}}} ,$$

12 $$- \frac{{{\text{d}}C_{{{\text{B}}_{{1}} }} }}{{{\text{d}}t}} = \alpha \times I_{{\text{A}}} \times \Phi_{{\text{A}}} \times F_{{\text{A}}} - \;I_{{{\text{B}}_{{1}} }} \times \Phi_{{{\text{B}}_{{1}} }} \times F_{{{\text{B}}_{{1}} }} + k^{\prime}_{{1}} \times C_{{\text{A}}} - k^{\prime}_{{3}} \times C_{{{\text{B}}_{{1}} }} ,$$

13 $$- \frac{{{\text{d}}C_{{{\text{B}}_{{2}} }} }}{{{\text{d}}t}} = \left( {{1} - \alpha } \right) \times I_{{\text{A}}} \times \Phi_{{\text{A}}} \times F_{{\text{A}}} - \;I_{{{\text{B}}_{{2}} }} \times \Phi_{{{\text{B}}_{{2}} }} \times F_{{{\text{B}}_{{2}} }} + k^{\prime}_{{2}} \times C_{{\text{A}}} - k^{\prime}_{{4}} \times C_{{{\text{B}}_{{2}} }},$$

14 $$- \frac{{{\text{d}}C_{{\text{C}}} }}{{{\text{d}}t}} = I_{{{\text{B}}_{{1}} }} \times \Phi_{{{\text{B}}_{{1}} }} \times F_{{{\text{B}}_{{1}} }} + I_{{{\text{B}}_{{2}} }} \times \Phi_{{{\text{B}}_{{2}} }} \times F_{{{\text{B}}_{{2}} }} - \;I_{{\text{C}}} \times \Phi_{{\text{C}}} \times F_{{\text{C}}} + k^{\prime}_{{3}} \times C_{{{\text{B}}_{{1}} }} + k^{\prime}_{{4}} \times C_{{{\text{B}}_{{2}} }} - k^{\prime}_{{5}} \times C_{{\text{C}}},$$

15 $$- \frac{{{\text{d}}C_{{\text{D}}} }}{{{\text{d}}t}} = \;I_{{\text{C}}} \times \Phi_{{\text{C}}} \times F_{{\text{C}}} + k^{\prime}_{{5}} \times C_{{\text{C}}} ,$$

16 $$- \frac{{{\text{d}}C_{{{\text{TOC}}}} }}{{{\text{d}}t}} = \;k^{\prime}_{{1}} \times C_{{\text{A}}} + k^{\prime}_{{2}} \times C_{{{\text{B}}_{{1}} }} + k^{\prime}_{{3}} \times C_{{{\text{B}}_{{2}} }} + k^{\prime}_{{4}} \times C_{{\text{C}}} + k^{\prime}_{{5}} \times C_{{\text{D}}} ,$$

where *α* denotes the photolytically degraded fraction of 4-chlorophenol to group B_1_ and *C*_i_ denotes the concentration of each lump i. The change in toxicity over time can be predicted by studying the toxicities exhibited by each lump. The concentration at any given time and the average toxicity of each group was predicted by preliminary calculations and validated from results reported in the literature (Table 1). Group D was excluded from the calculations as mineralised CO_2_ and water were present in the system. It did not contributed to the overall toxicity of the system. Toxicity was determined by Eq. (17), which was derived from Eq. (19). The equation derived to determine the toxicity of the system takes into account the fraction of unknown compounds belonging to group B_1_. The following equation was proposed to determine the toxicity of the system based on the above assumptions^24^:

17 $$- \frac{{{\text{d}}Toxicity}}{{{\text{d}}t}} = {k'_{\text{1}}} \times \frac{{{C_{\text{A}}}}}{{E{C_{{\text{50}}}}_{_{\text{A}}}}} + {k'_{\text{2}}} \times \left( {\frac{{{C_{{\text{B1}}}}}}{{E{C_{{\text{50}}}}_{_{{\text{B1}}}}}} + \frac{{{C_{{\text{B1unk}}}}}}{{E{C_{{\text{50}}}}_{_{{\text{B1}}}}}}} \right) + {k'_{\text{3}}} \times \frac{{{C_{{\text{B2}}}}}}{{E{C_{{\text{50}}}}_{_{{\text{B2}}}}}} + {k'_{\text{4}}} \times \frac{{{C_{\text{C}}}}}{{E{C_{{\text{50}}}}_{_{\text{C}}}}}$$

**Table 1:** Toxicity and photolytic properties of individual and grouped substances produced during the oxidation of 4-chlorophenol (UV/H_2_O_2_ oxidation mechanism)^36,38,42–44^.

Substance group	Compound	*EC*_50_ (mg L^−1^)		*ε* (M^−1^ cm^−1^)		*Φ* (mol Einstein^−1^)	
		Individual	Lump	Individual	Lump	Individual	Lump
A	4-chlorophenol	5.654	5.654	510.0	510.0	0.017	0.017
B_1_	Hydroquinone	0.088	0.0615	307.2	2044.9	0.047	0.025
	*p*-benzoquinone	0.035		13,612.0		0.025	
B_2_	4-chlorocatechol	5.932	6.892	381.3		0.057	0.0024
	Catechol	7.853		504.1		0.0001	
C	Maleic acid	3.852	63.154	769.9	1172.0	0.048	0.0351
	Fumaric acid	15.79		894.0		0.063	
	Malonic acid	12.28		697.0		0.27	
	Oxalic acid	817.2		630.9		0.15	
	Acetic acid	273.2		3159.2		0.028	
	Formic acid	235.6		504.1		0.006	
	Succinic acid	102.0		6300.0		0.0032	

### Validation of the kinetic model

The model proposed was based on the lumped mechanism described by Kusic et al.^36^ and Li et al.^45^. The kinetic, mass balance, and toxicity equations were solved using the Berkeley Madonna numerical calculation tool (Eqs. 10–17). The change in the concentration of each lump over time was determined using this method.

The simultaneous fitting of experimental and simulated data helped to determine the simulated photolytic, kinetic, and toxicity values of each lump. The determination of these values and the *EC*_*5*0_ values of the compounds can potentially help to predict the toxicity of wastewater during the UV/H_2_O_2_ treatment process, carried out under different operational conditions. The adjustment minimises the sum of squared residuals. The goodness of fit was determined by calculating the weighted standard deviation, *σ*, given by Eq. (18) as follows^30^:

18 $$\sigma = \sqrt {\frac{{\sum\limits_{{i = {1}}}^{N} {\left( {\frac{{C_{{{\text{exp}}}} - C_{{{\text{sim}}}} }}{{C_{{{\text{exp}}}} }}} \right)^{{2}} } }}{{N - {1}}}},$$

where *N* was the number of experimental values and *C*_exp_ and *C*_sim_ denote the concentration (of the compound or lump (i) determined experimentally and the concentration determined using the model, respectively. The data obtained from the direct photolysis experiments (*R* = 0) were used to determine the values of the photolytic parameters (quantum yield and extinction coefficient). The process of direct photolysis only affects the rate of disappearance of each lump. The photolytic contribution can be determined from the Lambert–Beer law. The initial values of the quantum yield (*Φ*) and extinction coefficient (*ε*) were presented in Table 1. After determining the photolytic parameters (Table 2), the experimental data were modelled in the presence of H_2_O_2_ to determine the values of the pseudo-first-order rate constants (*k*’_1_, *k*’_2_, *k*’_3_, *k*’_4,_ and *k*’_5_). The constants can be estimated by studying the reactions between the hydroxyl radicals and the lumps (except D; Eqs. 11–14). For the direct photolysis process (*R* = 0), the zero-order kinetic constants (*k*_i_) and the condition of total mass balance (Eq. 16) were considered for determining the experimental and theoretical concentrations of each group. The simulated constants were presented in Table 2. Following this, it was determined if the *EC*_50i sim_ values of each group (obtained by fitting the simulated toxicity data) agreed well with the experimentally obtained *EC*_50i exp_ values of each lump. The initial *EC*_50i_ values were determined using the *EC*_50_ values of the individual species reported in the literature^36,38,42–44^. The values obtained from literature reports have been presented in Table 1. It is worth mentioning that the toxicities exhibited by the substances (belonging to the same group) were similar (*EC*_50_ values of the same order of magnitude). This was the primary criterion of lumped modelling.

**Table 2:** Photolytic and toxicity properties determined using the proposed toxicity—kinetic model. Experimental conditions: *T* = 25 ℃; *C*_0_ = 200 mg L^−1^; pH = 6.0; Agitation = 700 rpm; *V*_reac_ = 1.7 L.

Group	*ε* (M^−1^ cm^−1^)	*Φ* (mol Einstein^−1^)	*EC*_50_ (mg L^−1^)
A	409	0.022	4.068
B_1_	480	0.015	0.075
B_2_	325	0.007	6.240
C	1485	0.005	73.300
D	–	–	–

High quantum yields were recorded for groups A and B_1_ (0.022 and 0.015 mol Einstein^−1^, respectively; Table 2). The data revealed that 4-chlorophenol could be easily degraded by the process of direct photolysis^41^. The branch mechanism is favoured when the photolytic pathway is the predominant mechanistic pathway. It was observed that a satisfactory adjustment between the simulated and experimental results could be achieved (σ ≅ 0.05) if a quantum yield value (for the reaction involving the photolysis of 4-chlorophenol), that is close to that reported by Pera-Titus et al.^46^ is used for the calculations. The quantum yield recorded for group C (*Φ = *0.005 mol Einstein^−1^), which consists of aliphatic compounds, agreed well with the quantum yield reported by Kralik et al. for the same group^40^.

As mentioned before, the theoretical toxicity of each group was represented as a function of the *EC*_50_ values of the identified species. The Microtox toxicity bioassay was used to determine the values presented in Table 2. The experimentally determined values were compared with the values presented in the literature (Table 1).

Table 3 presents the values of the kinetic constants determined using the proposed kinetic model. The value of *α* (the coefficient that correlates the production of substances (by photolytically degrading A) belonging to group B_1_ to the compounds belonging to group B_2_) was found to be 0.37. It must be remembered that the models comprised of compounds that exhibited similar toxicological properties. Group B_1_ comprised of all unknown aromatic compounds.

**Table 3:** Kinetic parameters predicted using the proposed toxicity—kinetic model at different H_2_O_2_/4-chlorophenol ratios. Experimental conditions: *T* = 25 ℃; *C*_0_ = 200 mg L^−1^; pH = 6.0; Agitation = 700 rpm; *V*_reac_ = 1.7 L.

Parameter	UV alone process			
*k*_1_ (s^−1^ mol L^−1^)	2.12 × 10^–5^			
*k*_2_ (s^−1^ mol L^−1^)	0.99 × 10^–5^			
*k*_3_ (s^−1^ mol L^−1^)	1.30 × 10^–5^			
*k*_4_ (s^−1^ mol L^−1^)	4.64 × 10^–5^			
*k*_5_ (s^−1^ mol L^−1^)	1.48 × 10^–5^			
*α*	0.37			
*σ*	0.054			

	UV/H_2_O_2_ process			
	*R* = 2	*R* = 20	*R* = 200	*R* = 400
*k*’_1_ (s^−1^)	2.46 × 10^–5^	1.51 × 10^–4^	6.87 × 10^–4^	2.05 × 10^–4^
*k*’_2_ (s^−1^)	1.07 × 10^–5^	1.16 × 10^–3^	1.25 × 10^–3^	1.19 × 10^–3^
*k*’_3_ (s^−1^)	1.54 × 10^–5^	5.83 × 10^–5^	1.23 × 10^–4^	8.56 × 10^–5^
*k*’_4_ (s^−1^)	4.96 × 10^–5^	1.65 × 10^–4^	1.14 × 10^–3^	4.44 × 10^–4^
*k*’_5_ (s^−1^)	1.67 × 10^–5^	4.50 × 10^–5^	9.10 × 10^–5^	6.82 × 10^–5^
*α*	0.35	0.21	0.16	0.19
*σ*	0.057	0.043	0.052	0.049

The results reveal that in the absence of oxidants, the highly toxic compounds, such as double-ringed chlorinated compounds (except *p*-benzoquinone and hydroquinone), were produced^47^. This was because, during the photolytic oxidation process, the formation of hydroquinone or *p*-benzoquinone was favoured over the formation of 4-chlorocatechol^36^. The experimental results presented in Table 3 validate this result. The coefficient *α*, determined using the simulation method, helps in understanding the nature of the unidentified products. The value of *α* (0.37) indicates that under these conditions, oxidation products that were more toxic than the parent pollutants were formed. Among all the identified compounds, hydroquinone was found to be present in the highest amount^48^. On the other hand, the value of this fraction decreased to 0.16 (at *R* = 200) when experiments were carried out in the presence of H_2_O_2_.

The values of the kinetic constants indicate that *k*_1_ (2.12 × 10^–5^ s^−1^ mol L^−1^) was higher than *k*_2_ (0.99 × 10^–5^ s^−1^ mol L^−1^) (under conditions of all oxidant dosages). The differences between *k*’_1_ and *k*’_2_ increased as the peroxide concentration increases. At conditions of *R* = 200, this difference increaseed by two orders of magnitude (*k*’_2_ = 1.25 × 10^–3^ s^−1^ versus *k*’_3_ = 1.23 × 10^–4^ s^−1^; Table 3). These results reveal that the radical pathway promotes the formation of the constituents of group B_2_ (over the constituents of group B_1_). This result has been previously reported by other groups^35,40,49^. It has been hypothesised that when the oxidation reaction occurs in the presence of hydroxyl radicals, the hydroxylation reaction was favoured over the chlorination reaction. This was because the *para*-position of chlorophenol is highly susceptible to attack during the direct photolysis reactions. The photoreactivity of the chlorine atom is higher than that of the hydroxyl group. In the presence of peroxides, chlorophenols react rapidly under conditions of UV radiation^41^.

**Figure 5:**
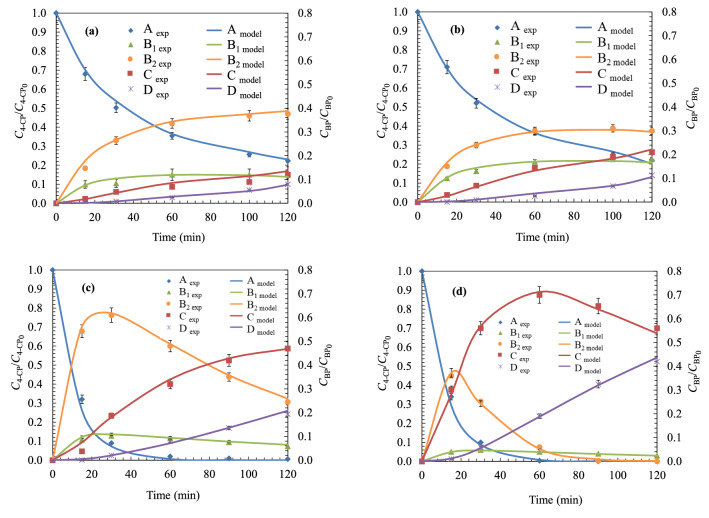
Evolution of the dimensionless concentration of each lump during the oxidation of 4-chlorophenol. Data fitted to the kinetic model at different doses of H_2_O_2_: (**a**) *R* = 0; (**b**) *R* = 2; (**c**) *R* = 20; (**d**) *R* = 200. Experimental conditions: *T* = 25 ℃; *C*_0_ = 200 mg L^−1^; pH = 6.0; Agitation = 700 rpm; *V*_reac_ = 1.7 L.

Under maximum dosage conditions, a large amount of 4-chlorocatechol was degraded and less amounts of *p*-benzoquinone (or hydroquinone) was produced. Similar results were obtained when the *k*’_1_ values at different H_2_O_2_ dosages were compared with each other. The value of *k*’_1_ increased as the value of *R* increase (from *R* = 2 (2.46 × 10^–5^ s^−1^) to *R* = 20 (1.51 × 10^–4^ s^−1^)). A slight drop in value was observed when *R* was in the range of 200–400 (*R* = 200 (6.87 × 10^–4^ s^−1^); *R* = 400 (2.05 × 10^–4^ s^−1^)). The amount of hydroxyl radicals present in the media (*R* ˃200) hindered the production of the compounds constituting group B_1_. Simultaneously, the formation of the compounds constituting group B_2_ was promoted (Fig. 5. The values of *k*’_1_ and *k*’_2_ indicated the efficiency with which 4-chlorophenol was degraded.

The results revealed that the values of the kinetic constants (*k*’_1_ and *k*’_2_) increase till *R* = 200 and become constant thereafter. It was observed that the amounts of degraded compounds did not increase with the increase in the amount of oxidant under conditions of constant radical concentration. A lower concentration of the generated hydroxyl radicals under steady-state conditions did not promote the efficiency of the reaction. The oxidant dosage under these conditions was known as the critical dose. When the critical *R*-value was reached, an increase in the peroxide dosage can potentially hinder the oxidation reaction, as the excess hydroxyl radicals can potentially react with H_2_O_2_ (scavenger reactions)^1,18^. Similar observations were made when the change in the concentration of 4-chlorophenol with time was experimentally studied under conditions of *R* = 20 (Fig. 5c) and 200 (Fig. 5d).

The critical dose under which the process of mineralisation or complete degradation was not promoted, was approximately 200 (*R* = 200). Although the reaction kinetics (direct oxidation of 4-chlorophenol) was not improved when the dosage was increased above 20, the analysis of the experimental results revealed that rate of mineralisation increased with the increase in the oxidant dosage. The concentration of the different oxidation products decreased at a higher rate under the experimental conditions. This was validated by the *k*’_4_ and *k*’_5_ values determined by the simulation experiments (Table 3). The values of the constants *k*’_4_ and *k*’_5_ indicated that when the dose of H_2_O_2_ was increased, the rate of mineralisation increased. This was reflected by the increase in the amounts of low molecular weight compounds (such as fumaric acid). The values of the constants *k*’_1_ and k’_2_, directly related to the primary degradation of *p*-chlorophenol, remained constant (*R* ≥ 20). However, the values of the other constants increased under similar conditions.

Aromatic compounds (constituents of groups B_1_ and B_2_) were the reactants in the second step of the oxidation reaction. The rate of the final step (group D; CO_2_ and H_2_O) rapidly increased at *R* = 200. The critical *R* for mineralisation was reached at dosages ˃ 200, though the maximum amounts of the compounds that belong to group B_2_ were formed within the first 20 min of the reaction. On the other hand, the amounts of compounds (carboxylic acids and low molecular weight compounds) constituting group C increased (Fig. 5d) till the first 60 min. Following this, the amounts of the compounds decreased as hydroxyl radicals (generated from H_2_O_2_) increased. These radicals could effectively react with these compounds to produce increased amounts of CO_2_ and H_2_O (group D).

**Figure 6:**
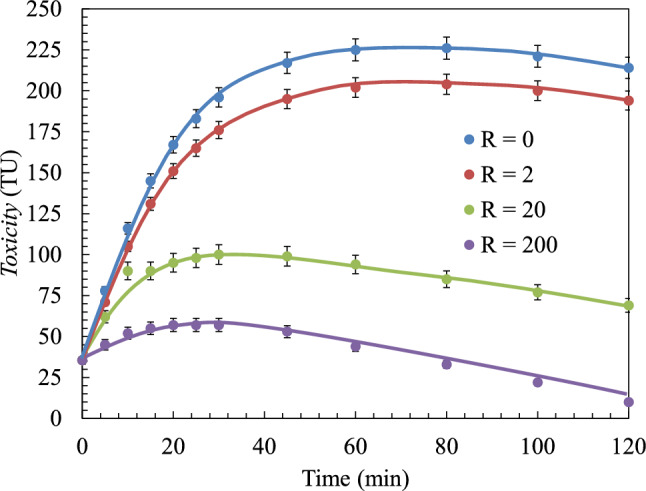
Prediction of the toxicity of the mixture during the oxidation of 4-chlorophenol (UV/H_2_O_2_ process) varying H_2_O_2_ dose.

Figure 6 showed the change in toxicity over time, determined using the kinetic model, at four different doses of peroxide during the process of photolysis. Good fitting results were obtained and the mean standard deviation was approximately 0.057 (*σ* ≅ 0.057). The toxicity was found to increase during the first 20 min of the reaction. Compounds belonging to group B_1_ (such as hydroquinone and *p*-benzoquinone) that were more toxic than 4-chlorophenol were produced under these conditions. An increase in the dose of H_2_O_2_ resulted in a decrease in the amounts of the compounds. The degradation compounds belonging to groups B_1_ and B_2_ were removed from the system under these conditions and by products (such as formic acid or fumaric acid) belonging to group C (less toxic compounds) were formed.

This increase in toxicity was also reported by Muñoz et al.^22^ and Karci et al.^23^ that they concluded that in the absence of oxidant or under conditions of inadequate oxidant dose, toxic substances (such as hydroquinone and *p*-benzoquinone) and condensation by products were formed. According to Ronco et al.^50^, a toxicity value of less than 1 TU indicated that the effluent was non-toxic or exhibited low toxicity. The values in the range of 1–10 TU correspond to slightly toxic effluents. When the value ˃11 TU, the effluent was considered to be highly toxic. At *R* = 200, a value of 9.95 TU was recorded for the wastewater under study. This sample could be used for biodegradation. The degradation pathway led to the formation of the compounds constituting group B_1_ under conditions of inadequate doses of oxidants. Under these conditions, the original wastewater sample was found to be more toxic and the chemical degradation pathway could not be complemented by a biological process.

## Methods

### Reagents

4-Chlorophenol (ClC_6_H_4_OH, Sigma-Aldrich, ≥ 99%), hydrogen peroxide (H_2_O_2_, Labkem, 30%, (v/v)), hydrochloric acid (HCl, Sigma-Aldrich, 33%), sodium hydroxide (NaOH, Labkem, ≥ 97.0%), sodium sulfite (Na_2_SO_3_, Sigma-Aldrich, 58.5%), *ortho*-phosphoric acid (H_3_PO_4_, Merck, 85%), and HPLC-grade methanol (CH_3_OH, Acros Organics, > 99.99%) were used as received. The following were used as calibration standards: 4-chlorocatechol (ClC_6_H_3_(OH)_2_, Sigma-Aldrich, 97%), hydroquinone (Supelco, certified reference material), benzoquinone (Supelco, certified reference material), pyrocatechol (Fluka Chemika,  ≥ 99%), maleic acid (HO_2_CCH=CHCO_2_H, Acros Organics,  ≥ 99%), fumaric acid (HOOCCH=CHCOOH, Fluka Chemika, 99.5%), and formic acid (CH_2_O_2_, Acros Organics, 99.0%). Deionised water was collected from a Milli-Q water purification unit supplied by Merck.

### Analytical techniques

The intermediates produced during the oxidation of 4-chloropehnol were detected using the high-performance liquid chromatography (HPLC) technique. The Waters Alliance 2695 system (WATERS, Milford, CT, USA) equipped with the Waters 2487 Dual λ Absorbance Detector (Waters, Milford, CT, USA) was used for the same. An INERTSIL ODS-3 column (150 mm × 4.6 mm, 5 μm) (GL Sciences, Torrance, CA, USA) was used to detect 4-chlorophenol (CP) and 4-chlorocatechol (4Cl-CC). The methanol:water (30:70, v/v) solvent system buffered with *o*-H_3_PO_4_ at pH 3 was used as the mobile phase. The flow rate was maintained at 1.0 mL min^−1^ and the sample volume was 1.0 µL. The analytes were detected at 280 nm. Other aromatic compounds such as hydroquinone (HQ), benzoquinone (BQ), and pyrocatechol (CC) were detected under the same conditions. The methanol:water (20:80, v/v) solvent system was used as the mobile phase for eluting the aromatic compounds. The experiments to detect BQ were carried out at a wavelength of 245 nm.

The aliphatic substances (maleic acid, fumaric acid, acetic acid, and formic acid) were separated on the INERTSIL ODS-4 column (250 mm × 4.6 mm, 5 μm) (GL SCIENCES, Torrance, CA, USA). The mobile phase consisted of (NH_4_)_2_HPO_4_ (20 mM) at pH 2. The compounds were detected at a wavelength of 214 nm. The flow rate was maintained at 1.0 mL min^−1^.

The concentration of unreacted H_2_O_2_ was determined spectrophotometrically at 420 nm, following the procedure outlined by Eisenberg^51^. The variation in the colour intensity was determined by recording the absorbance at 455 nm^52^. The rupture of the aromatic ring was detected by recording the absorbance of the sample at 254 nm using the PerkinElmer Lambda 10 UV/Vis spectrophotometer (Perkinelmer España, Madrid, Spain)^9^. The extent of oxidation was determined as the difference of the initial and final total organic carbon (TOC) using the Shimadzu TOC-VCSN analyser (Izasa Scientific, Alcobendas, Spain) to determine the efficiency of the water treatment process.

The variation in toxicity over time was determined by conducting a MICROTOX toxicity test (ISO 11348-3 (1998); Water Quality—Determination of the inhibitory effect of water samples on the light emission of *Aliivibrio fischeri* (Luminescent bacteria test)—Part 3: Method using freeze-dried bacteria^53^) using a Microtox SDY 500 Analyzer (Microbics Corp., New Castle, DE, USA). The reported method takes into account the changes in the luminescence emission of the luminescent *Aliivibrio fischeri* bacteria when it was exposed to toxic compounds. Toxicity values were expressed in terms of their inhibitory concentration (*IC*_50_) or variant effective concentration (*EC*_50_) (from the initial concentration of the pollutant (*C*_i_); the dilution necessary to reduce 50% the initial luminescence of the bacteria). These were most commonly expressed in toxicity units (TU) as follows (Eq. 19)^24,26^:

19 $$Toxicity = \frac{{{100}}}{{IC_{{{50}}} }} = \frac{{C_{i} }}{{EC_{{{50}}} }} .$$

All samples were treated with excess Na_2_SO_3_ to remove the residual H_2_O_2_ present in the system before analysing their toxicities. The samples were aerated to convert the residual sulfite to sulfate^38^.

All numerical calculations were performed using the Berkeley Madonna software (version 8.0; UC Berkeley, Berkeley, CA, USA). This software can be used to efficiently solve differential equations to predict the variation in concentrations and toxicities of each group of species over time. In addition, the program used the trial-and-error method to fit experimental and theoretical data to obtain the values of the photolytic, kinetic, and toxicological parameters of grouped substances. The method has been explained in the forthcoming sections.

### Experimental set-up

**Figure 7:**
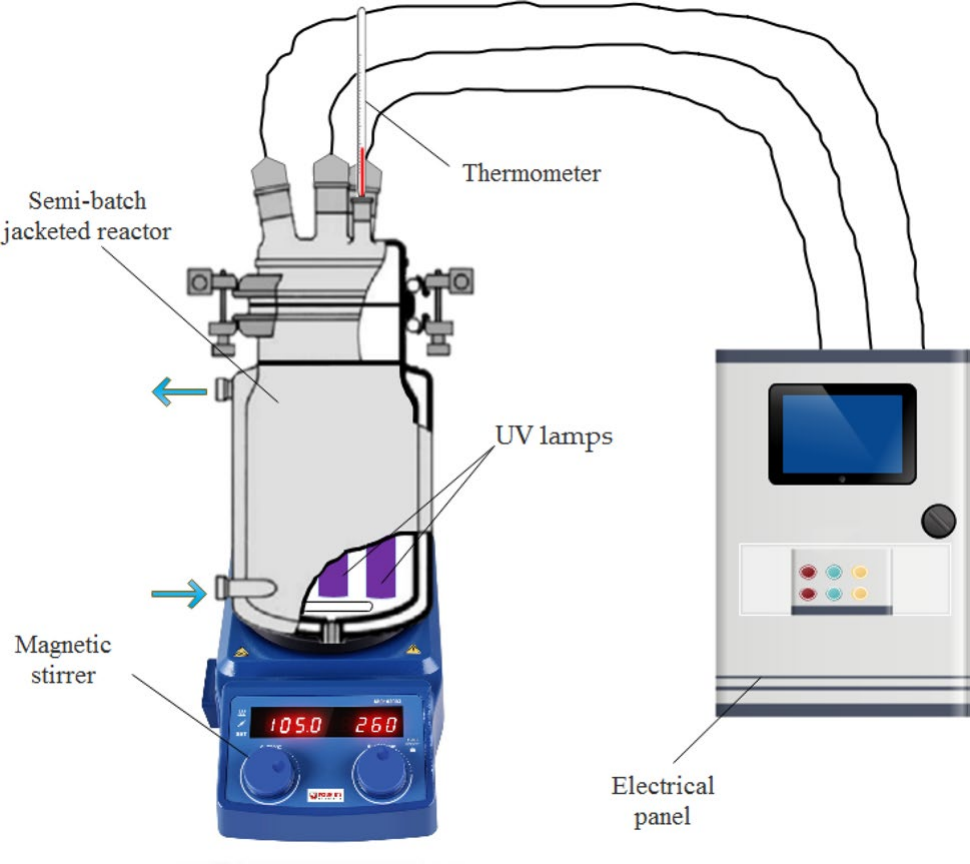
Experimental set‐up used to carry out photolytic assays.

Experiments were performed in a photochemical reactor (2 L batch mode) consisting of a Pyrex mixing vessel. The inner diameter was 100 mm and the height was 270 mm (Fig. 7). The temperature was maintained at 25 ℃ using a jacketed water recirculating thermostatic system. Three low-pressure mercury UV-C lamps (254 nm) that consume a nominal power of 8 W (Philips TUV 8 W G8T5, Philips, Madrid, Spain) were used for irradiation. UV irradiation was used instead of a visible or solar light source because these two sources emit only 5% of the UV irradiation required for the decomposition of hydrogen peroxide. The lamps were equidistant from each other and placed at the centre of the reactor. Each of the lamps was placed inside a quartz tube. Actinometrical measurements, revealed that the incident photonic flux (*I*_0_) was 3.17 × 10^–5^ Einstein s^−1^. The effective radiation path (*L*) was 5.85 cm.

### Experimental procedure

4-chlorophenol was dissolved in water (1.7 L) to obtain a solution of concentration 200 mg L^−1^. The solution was placed in the reactor and the mixture was stirred using a magnetic stirrer to produce a homogenous solution. Following this, the thermostatic bath was switched on to allow the solution to reach a temperature of 25 ± 1 ℃. The initial ratio of H_2_O_2_ to 4-chlorophenol (molar ratio (*R*); UV/H_2_O_2_ process) ranged from 2 to 400. The initial pH was adjusted as needed. The control sample was collected after adequately homogenising the solution (homogenisation time: 5 min). Subsequently, the UV-C lamp was switched on and the samples were collected at different time intervals. During the reaction, the temperature of the thermostatic bath was regulated such that the final temperature of the solution was 25 ± 1 ℃. All the experiments were conducted thrice. The average standard deviation was less than 5%.

## Conclusions

A kinetic model has been proposed to study the changes in the concentration and toxicity during the oxidation process of 4-chlorophenol by UV/H_2_O_2_. The effect of peroxide dose on degradation was studied to establish the operational conditions that promote the formation of non-toxic or slightly toxic effluents that can be subsequently treated via a biological pathway. The efficiency of the proposed model was calibrated using the experimental data, checking that the mean standard deviation was ˂0.05.

Based on the identification and analysis of the degradation products, two possible oxidative pathways were proposed, which occur simultaneously. The dose of the oxidant used and the reaction time indicated which of the two pathways would be predominantly operative. The main oxidation intermediates were grouped in different lumps based on various parameters: toxicity, extinction coefficient, and quantum yield.

The model allowed the determination of the kinetic and photolytic parameters. The estimation of these constants allowed the determination of the conditions under which more toxic and recalcitrant by products were formed. Conditions under which less toxic and easily degradable compounds were formed were also determined.

A less toxic effluent (9.95 TU) was produced at a pH of 6.0 when the dose of H_2_O_2_ was 200 (*R* = 200). Under these conditions, 4-chlorophenol could be degraded completely within 120 min from the start of the reaction. The percentage of mineralisation was approximately 40% and a total loss in colour was observed. This model can be potentially used in industries for the prediction of the operational behaviour of the UV/H_2_O_2_ system under different working conditions. The model can be used in association with a biological system. Moreover, the behaviour in terms of intermediate formation and toxicity can serve as a reference for other advanced oxidation techniques, including photocatalytic ones, with primary degradation and mineralisation rates similar to those studied here.

## Supplementary Information

Supplementary Information.

## Data Availability

The datasets generated during and/or analysed during the current study are available from the corresponding author on reasonable request.

## List of symbols

Agitation: Stirring power, rpm
*C*_0_: Initial concentration of 4-chlorophenol, mol L^−^^1^
*C*_exp_: Concentration of a compound or lump i measured experimentally, mol L^−^^1^
*C*_i_: Concentration of each lump i, mol L^−^^1^
*C*_j_: Concentration of a given compound present in solution, mol L^−^^1^
*C*_HO_·: Concentration of hydroxyl radicals, mol L^−^^1^
*Colour*_0_: Initial colouration was attributed to the 4-chlorophenol in solution, AU
*C*_sim_: Concentration of a compound or lump i obtained from the model, mol L^−^^1^
*C*_TOC_: Concentration of total organic carbon, mol L^−^^1^
*EC*_50_: Half maximal effective concentration, mol L^−^^1^
*EC*_50i_: Average half maximal effective concentration, mol L^−^^1^
*EC*_50i exp_: Experimentally determined average half maximal effective concentration, mol L^−^^1^
*F*_i_: Fraction of UV radiation absorbed by compounds of lump i, dimensionless
*I*_0_: Intensity of the emitted UV radiation, Einstein min^−^^1^
*IC*_50_: Half maximal inhibitory concentration, mol L^−^^1^
*k*’_1_, *k*’_2_, *k*’_3_, *k*’_4_ and *k*’_5_: Pseudo-first-order kinetic constant of each lump, s^−^^1^
*k*_i_: Zero-order kinetic constant (oxidation), s^−^^1^ mol^−^^1^ L
$\overline{k}_{{\text{i}}}$: Second order kinetic constant, s^−^^1^ L mol^−^^1^
*L*: Effective path length of the photoreactor, m
*N*: Number of experimental values
*R*: Peroxide/4-chlorophenol molar ratio, dimensionless
*t*: Time, s
*T*: Temperature, ℃
*Toxicity*: Toxicity, TU
*V*_reac_: Volume of dissolution, L

## Greeks

*ε*: Molar extinction coefficient, M^−^^1^ m^−^^1^
*α*: Fraction of all photolytically degraded 4-chlorophenol (group B_1_)
*σ*: Weighted standard deviation
*Φ*_i_: Quantum yield for lump i, mol Einstein^-1^

## Subscripts

0: Refers to a time or initial state
A: Refers to the lump formed by 4-chlorophenol
B_1_: Refers to the lump formed by hydroquinone, *p*-benzoquinone and other unknown that are more toxic than 4-chlorophenol
B_2_: Refers to the lump formed by 4-chlorocatechol, catechol, and other unknown compounds that are less toxic than 4-chlorophenol
C: Refers to the lump formed by formic acid, acetic acid, oxalic acid, and other compounds of similar nature
D: Refers to the lump formed by CO_2_ and H_2_O
exp: Refers to an experimentally obtained parameter
i: Refers to a certain lump i
j: Refers to the different compounds present in the solution
sim: Refers to a parameter obtained from the kinetic model
