# Supplementary material for: Kinetic modelling for concentration and toxicity changes during the oxidation of 4-chlorophenol by UV/H2O2
Source: Sci Rep. 2021 Aug 3;11:15726. doi: 10.1038/s41598-021-95083-7 (PMC8333101; doi:10.1038/s41598-021-95083-7)
Supplement: Supplementary file 1 — Supplementary Information. [file 41598_2021_95083_MOESM1_ESM.docx]

Supplementary Information

**Kinetic modelling for concentration and toxicity changes during the oxidation of 4-chlorophenol by UV/H_2_O_2_**

Cristian Ferreiro ^1,^*, Josu Sanz ^2^, Natalia Villota ^3^, Ana de Luis ^4^ and

José Ignacio Lombraña ^1^

^1^ Department of Chemical Engineering, Faculty of Science and Technology, University of the Basque Country UPV/EHU, Barrio Sarriena s/n, 48940 Leioa, Spain; ji.lombrana@ehu.eus

^2^ Department of Mathematics and Science Didactics, Faculty of Education, Philosophy and Anthropology of Donostia-San Sebastián, University of the Basque Country UPV/EHU, Barrio Sarriena s/n, 48940 Leioa, Spain; josu.sanz@ehu.eus

^3^ Department of Chemical and Environmental Engineering, Faculty of Engineering Vitoria-Gasteiz, University of the Basque Country UPV/EHU, Nieves Cano, 12, 01006 Vitoria-Gasteiz, Spain; natalia.villota@ehu.eus

^4^ Department of Chemical and Environmental Engineering, Faculty of Engineering in Bilbao, University of the Basque Country UPV/EHU, Plaza Ingeniero Torres Quevedo, 1, 48013 Bilbao, Spain; ana.deluis@ehu.eus

***** Correspondence: cristian.ferreiro@ehu.eus

**Table S1.** Reported methods of treating industrial wastewater containing 4-chlorophenol.

| **Advanced oxidation technology** | **Notes** | **Reference** |
| --- | --- | --- |
| Photo-Fenton | In the first stage, a heterogeneous reaction occurs on the surface of iron. In the second stage, a homogeneous reaction (Fenton reaction) is observed. The first stage proceeds through a radical pathway where 4‑chlorocatechol is one of the reactants (the hydroxyl radical attacks the substrate). The second stage involves the participation of *ortho*‑parachlorophenolperoxyl radicals and O_2_. | 1 |
| UV/TiO_2_ | It has been observed that the increase in the size of the TiO_2_ crystals (effect of the calcination temperature) favours the removal of 4‑chlorophenol. | 2 |
| UV/ZnO | The dependence of pseudo-zero-order kinetics on the different operational variables for the removal of 4-chlorophenol was investigated. It has been observed that inorganic anions such as Cl^-^ and SO_4_^2-^, common in water, act as blockers of the active centres. The proposed reaction mechanism involves an *o*-hydroxylation step that leads to the formation of high amounts of catechol. | 3 |
| UV/O_3_/H_2_O_2_ | Ozone decomposition results in the formation of hydroxyl radicals. The presence of the hydroxyl radicals did not result in an increased rate of oxidation at pH = 9. The values of the pseudo-first-order kinetic constants (4‑chlorophenol degradation reaction) estimated in the pH range of 7–12 were not significantly different from each other. | 4 |
| UV/TiO_2_/H_2_O_2_ | Photocatalytic processes carried out with a combination of UV light and H_2_O_2_ were less efficient (in producing mineralised compounds) than the UV/O_3_ and UV/H_2_O_2_ processes. A significant effect was not observed. | 5 |
| UV/H_2_O_2_ | It has been found that the addition of H_2_O_2_ increases the photolytic reaction rate by an order of magnitude. The extent of mineralisation of 4‑chlorophenol is improved and degradable compounds are produced in large amounts. | 6 |

**References**

1. Zhou, T., Li, Y., Ji, J., Wong, F.-S. & Lu, X. Oxidation of 4-chlorophenol in a heterogeneous zero valent iron/H_2_O_2_ Fenton-like system: Kinetic, pathway and effect factors. *Separation and Purification Technology* **62**, 551–558 (2008).

2. Sharma, S., Mukhopadhyay, M. & Murthy, Z. V. P. Treatment of Chlorophenols from Wastewaters by Advanced Oxidation Processes. *Separation & Purification Reviews* **42**, 263–295 (2013).

3. Gaya, U. I., Abdullah, A. H., Zainal, Z. & Hussein, M. Z. Photocatalytic treatment of 4-chlorophenol in aqueous ZnO suspensions: Intermediates, influence of dosage and inorganic anions. *Journal of Hazardous Materials* **168**, 57–63 (2009).

4. Pera-Titus, M., Garcı́a-Molina, V., Baños, M. A., Giménez, J. & Esplugas, S. Degradation of chlorophenols by means of advanced oxidation processes: a general review. *Applied Catalysis B: Environmental* **47**, 219–256 (2004).

5. Ruppert, G., Bauer, R. & Heisler, G. UV-O_3_, UV-H_2_O_2_, UV-TiO_2_ and the photo-Fenton reaction - comparison of advanced oxidation processes for wastewater treatment. *Chemosphere* **28**, 1447–1454 (1994).

6. Çatalkaya, E. Ç., Bali, U. & Şengül, F. Photochemical degradation and mineralization of 4-chlorophenol. *Environ Sci & Pollut Res* **10**, 113–120 (2003).
